# Conformational rearrangement of the NMDA receptor amino-terminal domain during activation and allosteric modulation

**DOI:** 10.1038/s41467-021-23024-z

**Published:** 2021-05-11

**Authors:** Vojtech Vyklicky, Cherise Stanley, Chris Habrian, Ehud Y. Isacoff

**Affiliations:** 1 Department of Molecular and Cell Biology, University of California, Berkeley, CA USA; 2 Institute of Physiology of the Czech Academy of Sciences, Prague, Czech Republic; 3 Biophysics Graduate Program, University of California, Berkeley, CA USA; 4 Helen Wills Neuroscience Institute, University of California, Berkeley, CA USA; 5 Molecular Biology & Integrated Bioimaging Division, Lawrence Berkeley National Laboratory, Berkeley, CA USA

**Keywords:** Single-molecule biophysics, Ion channels in the nervous system

## Abstract

N-Methyl-D-aspartate receptors (NMDARs) are ionotropic glutamate receptors essential for synaptic plasticity and memory. Receptor activation involves glycine- and glutamate-stabilized closure of the GluN1 and GluN2 subunit ligand binding domains that is allosterically regulated by the amino-terminal domain (ATD). Using single molecule fluorescence resonance energy transfer (smFRET) to monitor subunit rearrangements in real-time, we observe a stable ATD inter-dimer distance in the Apo state and test the effects of agonists and antagonists. We find that GluN1 and GluN2 have distinct gating functions. Glutamate binding to GluN2 subunits elicits two identical, sequential steps of ATD dimer separation. Glycine binding to GluN1 has no detectable effect, but unlocks the receptor for activation so that glycine and glutamate together drive an altered activation trajectory that is consistent with ATD dimer separation and rotation. We find that protons exert allosteric inhibition by suppressing the glutamate-driven ATD separation steps, and that greater ATD separation translates into greater rotation and higher open probability.

## Abstract (web summary)

N-Methyl-D-aspartate receptors (NMDARs) activation involves closure of the GluN1 and GluN2 subunit ligand binding domains, which is regulated allosterically by the amino-terminal domain (ATD). Here, smFRET, used to monitor conformational rearrangements of the NMDAR ATD, reveals that glutamate binding to GluN2 subunits elicits two identical, sequential steps of ATD dimer separation that are regulated by protons.

## Introduction

The amino acid glutamate serves as a principal excitatory neurotransmitter in the mammalian central nervous system^1^. Ionotropic glutamate receptors (iGluRs) bind presynaptically released glutamate and mediate fast excitatory synaptic transmission. iGluRs are ligand-gated cation channels that can be divided into three structurally and functionally distinct classes: α-amino-3-hydroxy-5-methyl-4-isoxazolepropionic acid receptors (AMPARs), kainate receptors, and N-methyl-D-aspartate receptors (NMDARs)^2,3^. NMDARs are highly permeable for Ca^2+^ and blocked by extracellular Mg^2+^ in a voltage-dependent manner^4–6^. These features are unique among iGluRs and underlie NMDARs key role in synaptic plasticity^7–9^. Both hyperfunction and hypofunction of the NMDAR signaling have been associated with a number of neuropsychiatric disorders such as Alzheimer’s disease, Parkinson’s disease, schizophrenia, autism spectrum disorder, mood disorders, or epilepsy^10,11^. More recently, NMDAR has been found to be expressed on various types of cancer cells and NMDAR signaling has been implicated in tumor growth^12–14^.

NMDARs are obligatory heterotetramers composed of two GluN1 and two GluN2(A–D) and/or GluN3(A–B) subunits^15–18^. Each subunit contains an extracellular amino-terminal domain (ATD) and ligand-binding domain (LBD), a transmembrane domain, and an intracellular carboxyterminal domain^19,20^. Binding of glutamate and coagonist glycine (or D-serine) to the LBDs of GluN2 and GluN1 subunits, respectively, triggers conformational changes that open a cation channel in the transmembrane domain^21–23^. The GluN1 and GluN2 ATDs form a pair of heterodimers that contact the LBDs and regulate agonist potency, kinetics, and open probability (Po)^19,20,24–26^. Both positive (polyamines) and negative (Zn^2+^, ifenprodil and protons) allosteric modulators bind the ATD^2,27–29^.

Recent cross-linking, X-ray crystallography, and cryo-EM studies have provided snapshots of receptor conformations^28,30–37^. The unliganded Apo state has not yet been resolved. As a result, the activation rearrangements have been deduced from two kinds of comparisons: receptor liganded by full agonists in the presence and absence of negative allosteric modulators or receptor that is bound to agonist versus competitive antagonist. The first comparison leaves it unclear whether the rearrangements reflect activation or allosteric modulation and the second are subject to uncertainty about whether the antagonists are neutral and recapitulate the Apo state. Nevertheless, together these studies, along with MD simulations, suggest that activation involves two classes of ATD rearrangement: an increase in interdimer distance and a rotation parallel to the plane of the membrane^28,30–41^. However, not all the studies agree on these points and the sequence of rearrangements, role of individual subunits, and place in the activation pathway that is subject to allosteric modulation remains unknown.

Cryo-EM has provided NMDA receptor structures of receptors with stabilizing mutations, truncations, and/or ligands that limit the number of observable states^42,43^. In contrast to these static images that still lack the beginning (Apo) and end (fully open channel) of the activation pathway^44^, electrophysiology has provided dynamic measures of transitions between functional states on the full-length native receptor, but limited to direct observation of the closed/open transition. To bridge the gap between structural analysis of individual conformations and electrophysiological analysis of functional transitions, we used single-molecule fluorescence resonance energy transfer (smFRET) as a spectroscopic ruler to analyze conformational dynamics of the activation pathway in functional, full-length, wild-type receptors^45^.

smFRET makes it possible to follow conformational rearrangements in real time. This analysis enabled us to observe single receptors stably occupying the resting conformation in the absence of agonist, the activated conformation in saturating agonist, and multiple transitions between these and stable intermediates at intermediate agonist concentrations, to observe the same behavior in a large number of receptors, and to relate the concentration dependence of state occupancy to that established in patch-clamp studies. FRET analysis on the single-channel level enables us to measure dwell times in specific conformations, analogous to single-channel patch-clamp recordings, but in this case directly detecting transitions between purely nonconductive states. We observe the relative position of the GluN1 N termini in the Apo state, find that GluN1 and GluN2 operate distinctly and cooperate during receptor activation, derive a kinetic model of the glutamate-driven ATD rearrangements, and address the mechanism of a key form of allosteric regulation. Our results suggest that glutamate specifically drives ATD dimer separation and that glycine allows the glutamate-driven rearrangement to take an altered trajectory that could be explained by rotation. Allosteric modulation of dimer separation translates into changes in the altered trajectory that can explain the influence on channel gating.

## Results

**Fig. 1:**
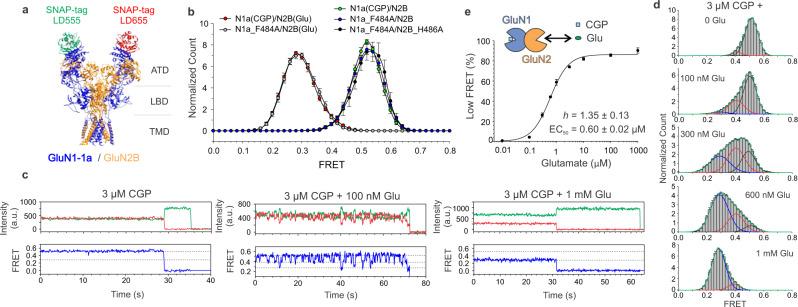
Glutamate alone triggers two sequential steps of multidomain rearrangement. **a** Representation of SNAP_GluN1-1a/GluN2B fusion protein construct (C-termini are present but not shown; Protein Data Bank accession 3KZZ [10.2210/pdb3KZZ/pdb] and 5IOU [10.2210/pdb5IOU/pdb]). SNAP tags are randomly labeled by a mixture of donor (green) and acceptor (red) synthetic dyes. smFRET conformational readout of NMDAR ATD interdimer distance is obtained from receptors with one donor and one acceptor dye. **b** CGP is a neutral GluN1 ligand and mimics the Apo state of the receptor. GluN1-1a(F484A) and GluN2B(H486A) mutations decrease glycine and glutamate affinity by ~6000× and ~300×, respectively^56,71^. Each histogram *n* = 5 movies, SEM error bars. **c** Glutamate binding leads to increased ATD interdimer distance in two discrete steps. Representative donor (green) and acceptor (red) intensity traces and smFRET traces (blue) in the absence (top), intermediate 100 nM (middle), and saturating 1 mM (bottom) glutamate concentration. In all, 3 µM CGP prevents binding by trace glycine. **d** smFRET histograms in the presence of various glutamate concentrations. Blue, red, and black lines show global 3-component Gaussian fit of low, intermediate, and high FRET states, respectively. Green line shows the sum of all three components. Each concentration *n* = 5 movies, SEM error bars. **e** Glutamate dose–response curve in 3 µM CGP (inset cartoon depicts CGP holding GluN1 LBD open and glutamate occupancy of GluN2B LBD depending on concentration; *n* = 5 movies, SEM error bars). Low FRET Gaussian fit peak taken as a measure of the activated state. *h* Hill coefficient.

To understand the sequence of conformational states leading to NMDA receptor channel opening, and their allosteric modulation, we employed smFRET, whose dynamic readout of distance change between two points has been used to follow activation rearrangements in glutamate receptors^46–51^, complementing atomic-resolution structural analysis of individual conformations^28,30,31,33–35,37,52^. We studied conformational changes induced by agonists in receptors that included a GluN1-1a subunit fused to N-terminal SNAP tag and C-terminal HA tag (SNAP_GluN1-1a). We coexpressed SNAP_GluN1-1a with either GluN2B or GluN2A. HEK293T cells expressing fully functional receptors (Supplementary Fig. 1a) were labeled with equimolar amounts of FRET donor LD555-BG and acceptor LD655-BG membrane-impermeable dyes that attach covalently and selectively to the self-labeling SNAP tag (Fig. 1a and Supplementary Fig. 1b)^53^. Solubilized receptors were immunopurified using the single-step SimPull method^50,54^, yielding sparse display on a passivated coverslip bearing a low-density lawn of anti-HA antibody, enabling thousands of receptors to be visualized in the donor and acceptor emission channels in a single field of view, and hundreds of donor/acceptor-labeled spots representing single receptors to be studied simultaneously for up to several minutes before bleaching. Labeling and pulldown were highly specific (Supplementary Fig. 1c, d).

### The Apo state is a stable conformation with the GluN1 ATDs close together

Crystallography and Cryo-EM studies have not yet captured the Apo state of the NMDA receptor. This unliganded state is key to understanding of the activation pathway. We set out to first determine the behavior of this state. The challenge is that it is difficult to entirely remove glycine and glutamate from solutions, leading to the possibility that trace amounts would ligand either the GluN1 or GluN2 subunits. This is especially a concern for the GluN1a subunit, which has a sevenfold higher affinity for glycine than does the GluN2B subunit for glutamate^55^. It is worth noting that smFRET is significantly more vulnerable to background amino acid contamination compared to electrophysiology due to its sensitivity to even single-subunit activation. We examined two conditions that would prevent binding by trace glycine: wild-type GluN1a/GluN2B receptor in a saturating (3 µM) concentration of the GluN1 antagonist CGP 78608 (CGP) and GluN1a(F484A)/GluN2B, in which the GluN1a-binding site is mutated to lower glycine affinity by 6000-fold^56^ and make it insensitive to trace glycine. These two conditions yielded narrow indistinguishable smFRET distributions around a single FRET peak at both no added (nominally zero) glutamate and saturating (1 mM glutamate) FRET levels (Fig. 1b). The agreement between GluN1a(F484A)/GluN2B and GluN1a(CGP)/GluN2B in nominal zero glutamate suggests that this represents the distance between GluN1 ATDs in the Apo state of the receptor (see “Methods”). To further test this, we combined the low glycine affinity mutant of GluN1a with a GluN2B subunit carrying a glutamate-binding site mutation, GluN2B(H486A), which lowers affinity by 300-fold, preventing binding to trace glutamate. The smFRET distribution of GluN1a(F484A)/GluN2B(H486A) in the absence of added ligand overlapped closely with GluN1a(F484A)/GluN2B in the absence of added ligand and wild-type GluN1a/GluN2B in saturating CGP (Fig. 1b). Together, these observations show that the unliganded Apo state is characterized by a stable ATD interdimer distance with a FRET value of 0.52. To quantify ATD interdimer dynamics in the Apo state, we compared the FWHM of Gaussian fits to smFRET histograms of individual movies for GluN1a(F484A)/GluN2B receptors in the absence of ligands (Apo) and GluN1a/GluN2B receptors fully occupied by agonist (100 µM glycine + 1 mM glutamate). We found no significant difference (*P* = 0.472, *n* = 5, *t* test), showing that ATD interdimer distance in the Apo state is no more dynamic than in the receptor fully occupied by agonists. In contrast to our observation of stable Apo state ATD positioning, a recent study that measured FRET within the LBD observed relatively large fluctuations and a broad FRET distribution in the wild-type receptor in the absence of added agonist, leading to the proposal that the Apo LBD is dynamic^49^. While it is possible that the LBDs are more dynamic than the ATDs, that study did not take measures to prevent liganding by trace amino acids (as we did with affinity-lowering mutations and competitive antagonists), leaving the possibility that the observations reflected transitions between the Apo and liganded states, rather than a dynamic Apo state.

### Glutamate alone triggers a multidomain rearrangement

Recent structural studies have observed that full occupancy by agonists in both the GluN1 and GluN2 subunits stabilizes a conformation with the ATDs splayed apart relative to agonist-bound proton or ifenprodil-inhibited receptors^28,31,33^. However, it is not known if the allosterically inhibited agonist-bound and the Apo state are one and the same or if agonist binding to either GluN1 or GluN2 is sufficient to initiate ATD dimer separation. To address this, we set out to measure FRET in different concentrations of glutamate under conditions where GluN1 does not bind agonists. We did this in the wild-type receptor in the presence of CGP, which we showed above to function as neutral antagonist, as well as in GluN1a(F484A)/GluN2B, in which GluN1a has a low enough affinity to prevent liganding by trace glycine (Fig. 1b), thereby mimicking the Apo state of GluN1. We found that, in the absence of liganding by both glutamate and glycine, smFRET efficiency was high ~0.52 (Fig. 1b, c), whereas in saturating (1 mM) glutamate, with no glycine liganding, smFRET efficiency shifted to ~0.28 (Fig. 1b, c). Both of these conditions showed stable FRET values, with rare transition to another FRET level. In contrast, at intermediate glutamate concentrations, ATD dimers transitioned between three conformational states with FRET values of ~0.52, ~0.40, and ~0.28 (Fig. 1c, middle). smFRET histograms obtained over a range of glutamate concentrations (Supplementary Fig. 1e, f) were well fit (green line) by a global 3-component Gaussian fit with high (~0.52, black line), middle (~0.40, red line), and low (~0.28, blue line) FRET states (Fig. 1d). A glutamate dose–response plot of occupancy of the low (~0.28) FRET (glutamate-activated) state shows an EC_50_ = 0.60 ± 0.02 µM and *h* = 1.35 ± 0.13 (Fig. 1e). These observations show that, in the absence of activation of GluN1 by glycine, glutamate initiates two discrete steps of conformational change at the ATD level. These intermediates in the activation pathway cannot be observed in single-channel current recordings because they do not open the channel and they have not been seen in crystallographic or cryo-EM analyses.

### Kinetic modeling of glutamate binding

**Fig. 2:**
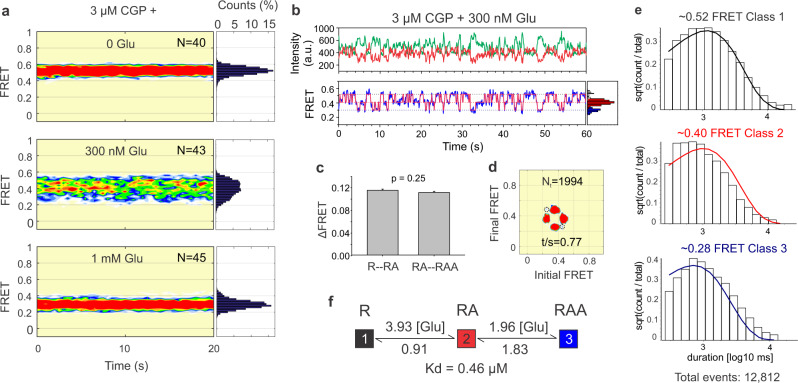
Kinetic modeling of glutamate binding. **a** Population FRET contour plots and its corresponding cumulative population histograms (on the right) for receptors treated by 3 µM CGP and 0, 300 nM, and 1 mM glutamate. Each contour plot contains FRET traces from one movie, *N* number of molecules. **b** Representative donor (green) and acceptor (red) intensity traces (top) and the corresponding smFRET trace (blue) with 3-state SKM idealization (magenta, bottom) in 3 µM CGP and 300 nM glutamate. Histograms, assigned to high, middle, and low FRET levels, are plotted on the right in black, red, and blue, respectively. **c** Glutamate binding increases ATD interdimer distance in two discrete steps whose $\triangle$FRET values are not significantly different (*P* = 0.25, two-tailed paired *t* test, *n* = 11, SEM error bars). **d** Transition-density plots from a representative movie show FRET values before (initial) and after (final) each transition (*t*/s = average number of transitions per second; *N*_t_ = total transitions per movie). Dotted circles indicate void at locations of direct transition between high and low FRET levels, indicating extreme rarity, i.e., transitions between high and low FRET go almost exclusively through the intermediate FRET state. **e** Dwell-time histograms for high, middle, and low FRET states fitted by kinetic model in (**f**). Total events indicate the total number of dwell times analyzed in all three histograms. **f** Kinetic scheme of glutamate binding to NMDAR. Resting (R), single-glutamate-binding site occupied (RA), both glutamate-binding sites occupied (RAA). The reaction mechanism illustrates association and dissociation rate constants (in µM^−1^ s^−1^ and s^−1^) that are consistent with two identical, independent binding events and a Kd similar to the activated-state glutamate EC_50_ dose –response (Fig. 1d).

A powerful method in single-channel patch-clamp analysis measures dwell times in different conductance states under different activation conditions to create a detailed kinetic model that describes the energetics of ligand binding, gating, and desensitization^57–60^. We applied this approach to analyze the pregating activation pathway at the ATD level by performing a kinetic analysis of glutamate-triggered rearrangements in the absence of glycine. smFRET traces in saturating 3 µM CGP (GluN1a in the Apo state) and 300 nM glutamate (near the glutamate EC_50_, Fig. 1e). smFRET traces were idealized by a 3-state model using the SKM algorithm (Fig. 2b and Supplementary Fig. 2b). FRET contour plots showed stability in FRET levels and occupancy over a 20-s observation time in zero-added 300 nM and 1 mM glutamate (Fig. 2a). Analysis of mean FRET levels from the idealization revealed similar FRET step sizes ($\triangle$FRET) for transitions between the high and medium FRET levels and between the medium and low FRET levels (Fig. 2c), suggesting two rearrangements of identical magnitude. Transition-density plots showed that the pathway from high to low FRET states was through the intermediate FRET state, and not direct (Fig. 2d and Supplementary Fig. 2a). These observations suggest that each of the FRET steps represents a conformational rearrangement triggered by glutamate binding to one of the two GluN2B LBDs.

Hidden Markov modeling provided a good fit to three states (Fig. 2e), corresponding to the FRET levels identified above (Fig. 1d), and organized in a linear two-step scheme (Fig. 2f). Alternate schemes either did not converge or produced kinetic models with very low-probability transitions that did not match the observations. In the successful model, the forward (activation) rate of the first step (from high to intermediate FRET) was twice that of the second step (intermediate-to-low FRET), and the same was true in reverse (deactivation), where the first step (low-to-intermediate FRET) was twice as fast as the second step (intermediate-to-high FRET) (Fig. 2f). This behavior suggests two independent, successive steps of glutamate binding to the GluN2B LBDs, triggering two identical ATD dimer separation rearrangements. The model predicts a glutamate dissociation constant (Kd = 0.46 µM) that is very similar to the observed EC_50_ (0.60 µM) (Fig. 1e), strongly supporting the model.

### Glycine-controlled ATD reorientation

**Fig. 3:**
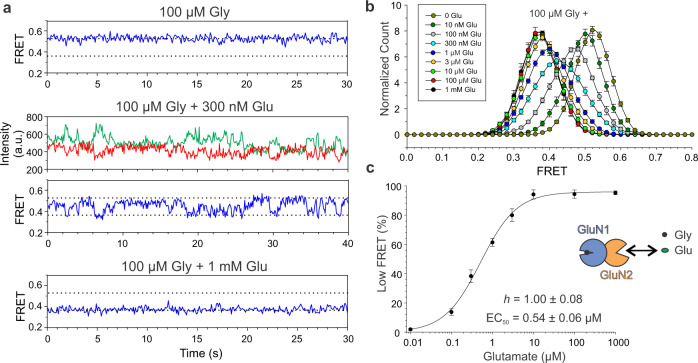
Glutamate dose–response curve in the presence of glycine. **a** Representative smFRET traces in the presence of 100 µM glycine (top), 100 µM glycine plus intermediate 300 nM glutamate (middle), and 100 µM glycine plus saturating 1 mM glutamate (bottom). **b** FRET histograms recorded with increasing glutamate concentration in the presence of 100 µM glycine. Each histogram *n* = 5 movies, SEM error bars. **c** Dose–response curve for glutamate in the presence of 100 µM glycine (*n* = 5 movies, SEM error bars).

**Fig. 4:**
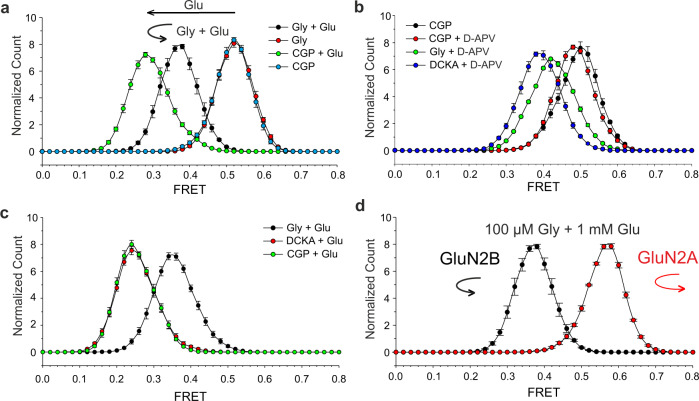
ATD dimer motion across activation states. **a** Glycine permits a different glutamate conformational pathway of activation. In all, 100 µM glycine alone (GluN1-closed_gly_/GluN2B-open_empty_, red) evokes no detectable FRET change (same as GluN1-open_CGP_/GluN2B-open_empty_, blue). In all, 100 µM glycine + 1 mM glutamate (GluN1-closed_gly_/GluN2B-closed_glu_, black) increases FRET compared to 1 mM glutamate alone (GluN1-open_CGP_/GluN2B-closed_glu_, green). Each histogram *n* = 5 movies, SEM error bars. **b** GluN2-competitive antagonist D-APV mimics an early stage of activation. GluN1-competitive antagonist DCKA increases ATD separation induced by D-APV indicating positive cooperativity between the GluN1 and GluN2B subunits. D-APV allows glycine to induce partial rotation (pH 8.5). Each histogram *n* = 5 movies, SEM error bars. **c** Neither CGP nor DCKA is capable of unlocking GluN1 to allow rotation (pH 8.5). Each histogram *n* = 5 movies, SEM error bars. **d** FRET histograms of SNAP_GluN1-1a/GluN2B (*n* = 5 movies, SEM error bars) versus SNAP_GluN1-1a/GluN2A (*n* = 5 movies, SEM error bars) receptors, in the presence of 100 µM glycine and 1 mM glutamate. GluN2A containing receptors exceeds the degree of rotation of the GluN2B in the presence of 100 µM glycine and 1 mM glutamate. Ligands were used at the following concentrations: 100 µM Gly, 1 mM Glu, 3 µM CGP, 100 µM DCKA, and 500 µM D-APV.

We next asked whether glycine binding to the GluN1 LBD also triggers a conformational change in the ATD and how liganding of GluN1 affects the glutamate-induced rearrangements. Remarkably, glycine alone had no effect on the observed GluN1 ATD interdimer distance: smFRET values were indistinguishable between 100 µM (saturating) glycine and 3 µM (saturating) CGP (Figs. 3a and 4a). In contrast, in 1 mM (saturating) glutamate, there was a substantial effect of glycine, however, rather than further decreasing FRET, as might be expected for a monotonic conformational pathway in which further activation would involve further ATD separation, Gly + Glu (GluN1-closed_gly_/GluN2B-closed_glu_) had higher FRET than CGP + Glu (GluN1-open_CGP_/GluN2B-closed_glu_) (Fig. 4a). This suggests that when glycine is bound, glutamate-induced ATD dimer motion takes a different conformational trajectory. Strikingly, the fully agonized GluN2A-containing receptor (GluN1-closed_gly_/GluN2A-closed_glu_), which reaches a fivefold higher open probability than GluN2B-containing receptors^18^, also reached a higher FRET level (Fig. 4d), suggesting that the degree of this altered motion determines the energetics of gating.

Together, these results suggest that glutamate alone drives ATD dimer separation (decreasing FRET) and that addition of glycine to glutamate permits rotation (increasing FRET from the glutamate-alone level), with greater rotation (higher FRET) yielding higher open probability. Glycine alone generates no change in FRET, either because of lack of ATD motion or isometric rotation. The low FRET (activated state) glutamate dose–response relation in glycine (EC_50_ = 0.54 + 0.06 µM, *h* = 1.00 + 0.08, Fig. 3c) was similar to that obtained in CGP with no glycine (Fig. 1e), suggesting that there is little influence of glycine on glutamate affinity. However, glycine shortened the occupancy times of the different FRET levels (Figs. 1c and 3a and Supplementary Figs. 2b and  3), indicating that it induces faster activation–deactivation rearrangements and/or increases the number or stability of intermediate states. In the presence of saturating concentrations of agonists (100 µM glycine + 1 mM of glutamate), we did not detect transitions to different FRET levels, suggesting that entry into desensitized states is not associated with changes in ATD interdimer distance (Fig. 3a and Supplementary Fig. 4b).

### Antagonist-induced ATD rearrangements

DCKA and D-APV are glycine- and glutamate-binding site competitive antagonists, respectively. A comparison of the recent Xenopus GluN1a/GluN2B structures in the presence of these antagonists versus agonist-bound forms shows large movements in the extracellular domains. But are DCKA and D-APV neutral antagonists that stabilize the open LBD/resting state, in the manner of CGP for GluN1a? We find that, unlike CGP, DCKA and D-APV both induce a partially activated conformation, providing a useful tool to study the early stages of activation. D-APV binding in the presence of CGP leads to a very small ATD dimer separation (from 0.50 to 0.48 FRET, *P* = 0.03, *n* = 5, *t* test). However, when DCKA substitutes for CGP in the GluN1-binding pocket, D-APV induces a much larger ATD dimer separation (0.38 FRET). When DCKA is replaced by glycine, so that the GluN1 LBD can fully close, D-APV produces sufficient GluN2 clamshell closure to allow the receptor to undergo a small rotation that brings the ATD dimers back into slightly closer proximity (0.42 FRET). This degree of rotation is too small to open the channel pore. These results imply that there is positive cooperativity between the GluN1 and GluN2B subunits in the early stages of NMDA receptor activation (Fig. 4b, pH 8.5). It is noteworthy that although DCKA supports a small amount of activation, in the presence of glutamate, the conformation is indistinguishable from that of CGP (Fig. 4c, pH 8.5), suggesting that this partial activation is insufficient to unlock the GluN1 subunit and allow for rotation, and consistent with lack of opening in glutamate + DCKA^61^.

### The proton-induced ATD rearrangement is state-dependent

**Fig. 5:**
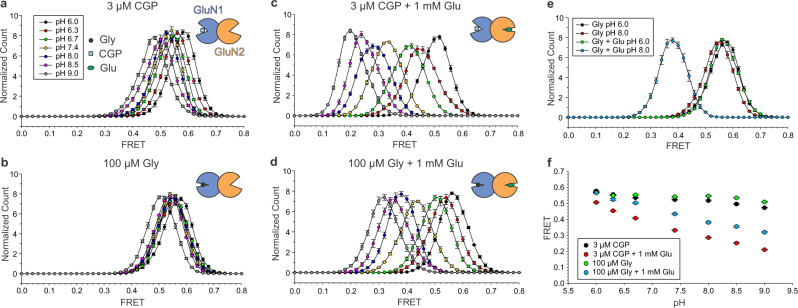
pH selectively modulates glutamate-induced dimer separation. **a**–**d** Effect of pH on the smFRET histogram of the SNAP_GluN1-1a/GluN2B receptor in 3 µM CGP (*n* = 5 movies for pH 6.0, 6.3, 6.7, 8.0, and 8.5, *n* = 4 movies for pH 7.4, 9.0, SEM error bars) (**a**), 3 µM CGP + 1 mM glutamate (*n* = 5 movies for pH 6.0, 6.7, 7.4, 8.5, and 9.0, *n* = 4 movies for pH 6.3, 8.0, SEM error bars) (**c**), 100 µM glycine (*n* = 5 movies for each pH condition, SEM error bars) (**b**), and 100 µM glycine + 1 mM glutamate (*n* = 5 movies for pH 6.3, 7.4, and 8.5, *n* = 4 movies for pH 6.0, 6.7, 8.0, and 9.0, SEM error bars) (**d**). **e** Little difference in smFRET histograms between pH 6.0 and 8.0 in 100 µM glycine (both histograms *n* = 5 movies, SEM error bars), but large difference in 100 µM glycine + 1 mM glutamate (both pH 6.0 and 8.0 histograms *n* = 4 movies, SEM error bars). pH 6.0 brings receptor fully occupied by glycine and glutamate (100 µM glycine + 1 mM glutamate) into a state indistinguishable from the resting Apo state at pH 8.0 (here represented by glycine-bound state). **f** Mean maximum FRET histogram values in various ligand combinations across pH.

Protons inhibit channel opening. It is not clear which steps in activation they affect. To study the modulation by protons, we obtained smFRET histograms in GluN2B-containing receptors over a range of proton concentrations, from pH 6.0 to 9.0, in various ligand combinations: CGP (GluN1-open_CGP_/GluN2B-open_empty_), Gly (GluN1-closed_gly_/GluN2B-open_empty_), CGP + Glu (GluN1-open_CGP_/GluN2B-closed_glu_), and Gly + Glu (GluN1-closed_gly_/GluN2B-closed_glu_). Across these different ligand conditions, decreasing pH shifted the FRET distribution to higher values (Fig. 5), consistent with previous structural observations and with the known negative allosteric modulation of GluN1/GluN2A by protons^28^. In the absence of glutamate, whether the GluN1 LBD was bound to agonist (glycine) or antagonist (CGP), the FRET shift with pH was very small (Fig. 5a, b) and there was almost no difference between pH 6.0 and 8.0 (Fig. 5f). In contrast, in the presence of glutamate, whether the GluN1 LBD was occupied by glycine or CGP, the effect of pH was large (Fig. 5c, d). In glycine +  glutamate, pH 6.0 brought the receptor ATD into a FRET state indistinguishable from the resting Apo state at pH 8.0, represented by the CGP-alone bound state (Fig. 5e, f). Finally, there was little effect of pH on the increase in FRET from the CGP-antagonized state to the glycine-agonized state in the presence of glutamate (Supplementary Fig. 5). These observations show that pH specifically controls the glutamate-induced ATD dimer separation branch of the activation pathway. The specificity of the modulation to this glutamate-driven preopening branch of the activation pathway explains why high pH alone cannot open the channel.

## Discussion

Structural and modeling studies have provided views of multiple conformations of NMDA receptors in different ligand states^28,30–36,38–41,52^. Structural analyses have not yet captured the Apo state and, so, estimated the activation rearrangement based on either release from allosteric inhibition of the fully agonized receptor or a comparison of the competitive antagonist- and agonist-bound forms. Several of these studies detected ATD dimer separation and subunit rotation^28,31,33^, although others observed only dimer separation and the degree of movement varied^30,33,37^. ATD dimer rotation was especially pronounced in the MD study where it reached ~45°^39^. Dolino and collegues^48^ and Durham et al.^49^ used smFRET to detect conformational changes in the TMD and within the LBDs, respectively. These intradomain measurements have provided valuable insight into conformational spread and dynamics within these domains and complement our GluN1 ATD interdimer readout of NMDAR domain rearrangements.

**Fig. 6:**
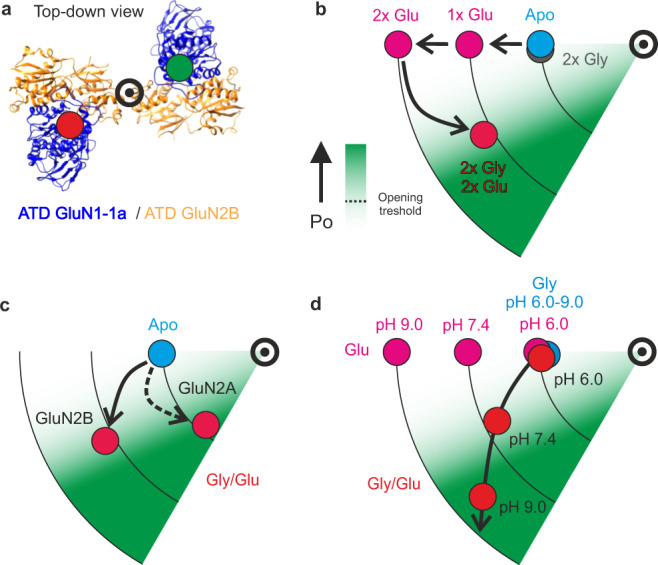
Schematic representation of ATD conformational transitions. **a** Top-down view of the ATD domain (5IOU [10.2210/pdb5IOU/pdb]). Red and green filled circles show approximate positions of the SNAP tags labeled by donor and acceptor dyes, respectively. Bullseye denotes the center of rotation. **b** Model of NMDAR activation by glycine and glutamate. **c** Model of NMDAR activation for GluN2B- and GluN2A-containing receptors. **d** Model of NMDAR modulation by pH in the presence of Gly, Glu, and Gly/Glu. Maximal degree of rotation (achieved in Gly/Glu, pH 9.0) corresponds to 45° rotation of ATD dimers after receptor activation^39^.

We used smFRET to obtain a real-time readout of receptor conformational dynamics in the full-length wild-type receptor, avoiding stabilizing modifications that could interfere with function (Fig. 6a). We obtained signatures of two phases of ligand-driven ATD motion: a first phase of ATD dimer separation and a subsequent different kind of motion that further activates the receptor but brings the ATDs closer together. We found that the ATD dimer separation is driven by glutamate alone in an early activation phase that has been inaccessible to prior dynamic analysis with patch-clamp since it does not open the channel. We show that ATD-dimer separation proceeds in two identical steps between close, intermediate, and distant conformations. Our kinetic modeling shows that the dynamics of the transitions between these conformations fit with two independent steps of glutamate binding (Fig. 6b), suggesting that each is driven by closure of one of the GluN2B LBDs. Although the kinetic scheme describes our data well, it is likely to be oversimplified.

This tight coupling between glutamate-triggered events (i.e., GluN2 LBD closure) and the rearrangement of the GluN1 ATDs is understandable given that the ATD of one GluN1 subunit crosses over to interact with the ATD of the neighboring GluN2 subunit, which in turn contacts that same GluN2’s LBD. It reinforces the notion that the ATDs and LBDs operate as a coordinate inter-subunit assembly.

In contrast to the rearrangements induced by glutamate, glycine alone evokes no detectable ATD rearrangement. However, when glycine is added to glutamate, FRET increases, indicating a decrease in ATD dimer separation. Since this addition of GluN1 agonism further advances the receptor down the activation pathway compared to GluN2 agonism alone, this coming together of the ATDs must represent a different kind of rearrangement. Consistent with this, the magnitude of this motion is greater in the GluN2A receptor, which has a higher probability of opening than the GluN2B receptor^18^.

Using cryo-EM, Tajima and colleagues^31^ identified three structural classes of the NMDA receptor in the presence of glycine and glutamate, and proposed these to represent two preopen states and the open state, or possibly desensitized state(s). Our results suggest that desensitization does not alter ATD distance and that two of the structural states (ATD distances of 23.7 Å and 36.4 Å) may correspond to our singly and doubly glutamate-bound states, respectively, while the third (ATD distance of 31.5 Å) could represent the rotated/activated state.

Cryo-EM structures in competitive antagonists have yielded conflicting pictures. In the GluN1 antagonist DCKA and GluN2 antagonist D-APV, Zhu and colleagues observed considerable heterogeneity in ATD configuration, with various dimer distances and degrees of ATD–ATD association^33^. In contrast, in the GluN1 antagonist L689,560 (L689) and GluN2 antagonist SDZ-220-040 (SDZ), Chou et al. observed a single dominant conformational class with no disruption of the ATDs and stable ATD–ATD association^37^. It is not clear if the differences between these studies in stability/dynamics arise from differences in the antagonists or other factors. Our results show a single-peak, narrow FRET distribution in the presence of both DCKA/D-APV and L689/SDZ, suggesting that the antagonist-bound state has a conformationally stable ATD. We found that receptor conformation, judged by the smFRET histogram peak value, in the presence of competitive antagonist 1 µM L689 and 50 µM SDZ, to be significantly lower than that in CGP at pH 8.5 (*P* = 0.023, *n* = 5, *t* test, Supplementary Fig. 4a), which was indistinguishable from the ATD interdimer distance in the Apo state. This suggests that competitive antagonists L689 and SDZ induce a small degree of LBD clamshell closure and advance the receptor partially along the activation pathway and can explain why, in their presence, Chou and colleagues are able to capture the receptor ATD and LBD in activated conformation^37^.

Interestingly, we find that, whereas CGP functions as a neutral agonist of GluN1, which stabilizes it in a state that is indistinguishable from the Apo state at the ATD domain level, DCKA is a weak agonist, too weak to support channel opening in the presence of glutamate, but sufficient to augment the small ATD separation induced by weak agonism due to D-APV. Thus, GluN1 glycine-binding site activation appears to be subdivided into two phases: (1) a phase where weak GluN1 activation (DCKA) helps Glu2B with ATD separation and (2) a phase where strong GluN1 activation (glycine) unlocks GluN1 for rotation.

Taken together, our observations suggest that GluN1 and GluN2 have very different roles in NMDA receptor activation and that activation and relief from proton inhibition follow similar ATD rearrangements. Glutamate binding to GluN2 drives a large radial rearrangement of the ATDs, but glycine binding to GluN1 produces little or isometric ATD movement. We propose that the unliganded GluN1 suppresses the rotation needed to open the channel and that glycine binding unlocks this to permit rotation. In this model, the GluN2 subunits drive two identical steps of early activation and the GluN1 subunits are permissive, allowing the high-energy glutamate-bound conformation to be converted to rotation upon glycine unlocking. The analog rotation is converted proportionately into a digital probability of opening of the channel gate.

The coupling between ligand binding and ATD motion can serve as a substrate for the regulation of gating by allosteric modulators that bind the ATD^2,27–29^. Protons inhibit NMDA receptors with an EC_50_ close to physiological pH ~7.4, yielding tonic inhibition and high sensitivity to pH change^62,63^. These pH changes can originate from protons coreleased with glutamate from acidic synaptic vesicles or as a result of pathological conditions such as ischemia or seizure^27,64^. The exact location of the proton sensor is still unclear; however, there is strong evidence that tonic proton inhibition is the fundamental mechanism through which ATD-binding allosteric modulators control NMDA receptor function^27,65^. This places proton-induced inhibition at the heart of both receptor activation and allosteric modulation. X-ray crystallography and Cryo-EM studies comparing structures with and without ATD-binding-negative allosteric modulators have shown that ifenprodil and protons reduce ATD interdimer distance, opposing the dimer separation that is induced by agonists^28,31,33^. Here we confirm this for protons in dynamic receptors. Our FRET readout of rotation (the increase in FRET of glutamate-bound receptors upon transition from CGP to glycine) indicates that rotation has little sensitivity to pH. Thus, negative allostery by protons appears to act specifically by suppression of the glutamate-induced step of ATD dimer separation. Strikingly at the highest proton concentration that we tested, pH 6.0, we find that the receptor enters a conformation that is indistinguishable from the Apo state (judged by the ATD interdimer distance), thus explaining the degree to which activation is suppressed. When rotation is possible in the presence of glycine, glutamate drives greater rotation at higher pH, enhancing open probability (Fig. 6d), consistent with the greater opening seen in GluN2A receptors compared to GluN2B receptors (Fig. 6c).

Our results show how specific steps of NMDA receptor domain reorganization, as seen at the ATD level, are individually controlled by the two coagonists glutamate and glycine and regulated by tonic proton inhibition. The observed positive cooperativity and distinct roles in receptor activation of the GluN1 and GluN2 subunits are intriguing in view of the recently discovered behavior of single-ligand heterotetrameric GluN3-containing excitatory glycine-gated NMDA receptors, which are activated by liganding of the GluN3 subunit and inhibited by liganding of the GluN1 subunit, with GluN1 producing a unique form of inactivation^66,67^. Our optical approach on functioning wild-type receptors provides a complement to cryo-EM that can verify structural observations, help resolve contradictory results, and fill in transition pathways between stable structural states. Our model provides a framework that could aid in the design of allosteric modulators to treat forms of cancer and neuropsychiatric disorders that are associated with NMDAR signaling dysfunction.

## Methods

### DNA constructs and site-directed mutagenesis

SNAP_GluN1-1a construct was made as follows: SNAP_f_ tag (SNAP) was inserted after C22 residue. To improve SNAP-ATD coupling, residues RLGKPGL were deleted from the SNAP C-terminus. Flexible linker followed by HA affinity tag was inserted right before GluN1-1a stop codon (GGGGS-YPYDVPDYA). The amino acid point mutations (including the SNAP-tag construct) are numbered according to the wild-type full-length protein, including the signal peptide, with the initiating methionine as 1.

### Cell culture and transfection

Human embryonic kidney 293T (HEK293T) cells were cultured in Opti-MEM supplemented with 5% fetal bovine serum (FBS) at 37 °C in 5% CO_2_. Cells were transfected at ~80% confluency with expression vectors containing rat full-length glutamate receptor subunits SNAP_GluN1–1a and GluN2A or GluN2B using Lipofectamine 2000. Equal amounts of cDNAs encoding for GluN1 and GluN2 subunits were used (900 ng cm^−2^ of cell culture). For patch-clamp experiments: cells were cotransfected with eGFP as a transfection marker (3:3:1 ratio; 150 ng cm^−2^). After 5 h of transfection, cells were trypsinized and reseeded at low density on poly-l-lysine-coated glass coverslips in Opti-MEM supplemented with 1.5% FBS, 20 mM MgCl_2_, 50 µM 5,7-dichlorokynurenic acid (DCKA), and 400 µM d,l-2-amino-5-phosphonopentanoic acid (d,l-APV). For smFRET and confocal experiments: DNA/lipofectamine mixture in Opti-MEM transfection media supplemented with 3% FBS, 20 mM MgCl_2_, 50 µM DCKA, 800 µM d,l-APV, and 20 µM ifenprodil was left on cells for 24 h.

### Patch-clamp electrophysiology

Experiments were performed 24–48 h after transfection. Whole-cell voltage-clamp recordings from HEK293T cells were made with a patch-clamp amplifier Axopatch 200B after a capacitance and series resistance (<10 MΩ) compensation of 80–90%. Patch pipettes (3–5 MΩ) were filled with an intracellular solution containing (in mM) 120 gluconic acid, 15 CsCl, 10 BAPTA, 10 HEPES, 3 MgCl_2_, 1 CaCl_2_, and 2 ATP-Mg salt (pH-adjusted to 7.2 with CsOH). Extracellular solution (ECS) contained the following (in mM): 160 NaCl, 2.5 KCl, 10 HEPES, 10 glucose, 0.2 EDTA, and 0.7 CaCl_2_ (pH-adjusted to 7.4 with NaOH). Experiments were performed at room temperature. Data were collected (low-pass filtered at 2 kHz and sampled at 10 kHz) and analyzed using pClamp 10.

### SNAP-tagged NMDAR labeling and solubilization

Transfected cells in 35-mm Petri dish were washed with extracellular solution (ECS) containing (in mM) 160 NaCl, 2.5 KCl, 2 CaCl2, 1 MgCl2, and 10 HEPES, pH 7.4, and labeled at room temperature with 1.5 μM donor LD555-BG and 1.5 μM acceptor LD655-BG in ECS for 25 min. Cells were washed twice with PBS and harvested in PBS containing 1 mM PMSF using a cell scraper. Cells were then pelleted by spinning at 5,000 g for 5 min and lysed in 120 µl of lysis buffer consisting of 150 mM NaCl, 20 mM Tris (pH 8.0), 1% lauryl maltose neopentyl glycol (LMNG), 0.1% cholesteryl hemisuccinate (CHS), protease inhibitor cocktail (Thermo Fisher Scientific), 1 mM PMSF, 5% glycerol, and 5 mM dithiothreitol (DTT). After 1.5 h of lysis at 4 °C, lysate was centrifuged at 16,000 × *g* for 20 min and the supernatant was collected and kept on ice. For experiments with nominal zero or subsaturating concentrations of glutamate, the lysate was washed 3× by 500 µl imaging buffer by pass through a 100-kDa spin column (MilliporeSigma) to reduce cell-originated background glutamate concentration in cell lysate before loading into smFRET imaging chamber.

### SimPull receptor isolation and surface display

To inhibit nonspecific protein adsorption, flow cells for single-molecule experiments were prepared using mPEG (Laysan Bio) passivated glass coverslips and doped with biotin PEG16^50^. Before each experiment, coverslips were incubated with 20 µg/ml NeutrAvidin (Thermo Fisher Scientific) for 15 min, followed by 1/100 biotinylated anti-HA antibody (Abcam, ab26228) for 1 h. The dilutions and washes to remove free reagents were done in T50 buffer (50 mM NaCl, 10 mM Tris, pH 7.4). Cell lysate incubation times (1–60 min) and dilutions in imaging buffer (1–10×) varied depending on receptor expression level to achieve sparse immobilization of labeled receptors on the surface. Unbound receptors were washed out of the flow chamber and the flow cells were then washed extensively (up to 75× the cell volume).

### smFRET measurements

Receptors were imaged for smFRET in imaging buffer consisting of (in mM) 160 NaCl, 2.5 KCl, 2 CaCl_2_, 10 MgCl_2_, 20 (MES, pH 6.0, 6.3, and 6.7; HEPES, pH 7.4, 8.0, HEPBS, pH 8.5, or CHES, pH 9.0), 50 glucose, 1 DTT, 0.01% LMNG, 0.001% CHS, 3 Trolox, and 2 protocatechuic acid. In all, 50 nM protocatechuate-3,4-dioxygenase was added into the imaging buffer immediately before it was loaded into imaging chamber. All buffers were made in UltraPure distilled water (Invitrogen). pH of imaging buffer was 8.0, if not stated otherwise. Different imaging buffers with pH in the range 6.0– 9.0 did not affect donor and acceptor dye performance in a way that would result in a significant change in γ factor. Samples were imaged on an objective-based (1.65 NA ×60 objective (Olympus)) TIRF microscope equipped with Photometrics Prime 95B sCMOS camera at 100-ms frame rate. Lasers at 532 nm (Cobolt) and 632 nm (Melles Griot) were used for donor and acceptor excitation, respectively. Micro-Manager 2.0-gamma was used for acquisition.

### smFRET data analysis

FRET efficiency was calculated as (*I*_A_ − 0.105 × *I*_D_)/(*I*_D_ + *I*_A_), in which *I*_D_ and *I*_A_ are the donor and acceptor intensity, respectively, after background subtraction and γ factor correction (γ = 1.1). Single-molecule intensity traces showing single-donor and single-acceptor photobleaching with a stable total intensity and FRET lasting at least 20 s were collected using SPARTAN 3.7.0^68^. smFRET histograms were compiled from the first 20 s of all smFRET traces passing criteria found in a single movie and normalized. Error bars in the histograms represent the standard error of mean from ≥4 independent movies. Each averaged histogram consisted of >100 molecules pooled from multiple movies. Each experiment was performed ≥3 times to ensure reproducibility, only one such experiment is presented here. Gaussian fitting to histograms from individual movies was done in Fityk 1.3.1. One Gaussian was fit to smFRET histograms to extract pH-induced conformational changes. For glutamate dose –response in the presence of glycine or CGP, two (0, 100 µM, and 1 mM glutamate) or three (10 nM–10 µM glutamate) Gaussians were fit to histograms and the area underneath the low FRET Gaussian was used as a measure of activated-state occupancy. Dose–response data were fit by 3-parameter Logistic function in SigmaPlot 10.0. Hidden Markov modeling was performed on full-length smFRET traces. Traces were idealized using the segmental K-means (SKM) algorithm in SPARTAN. Zero FRET state was removed from idealized data and the kinetic modeling was done in Qub 1.4 using maximum-likelihood fitting (MIL). About 200-ms dead time was applied.

### Overcoming glycine contamination

Because of the high affinity of NMDAR for glycine and the difficulty of avoiding trace glycine in solutions^69,70^, experiments without agonist on GluN1 were performed in zero-added glycine either in the presence of the glycine-binding site competitive antagonist CGP 78608 (CGP, 3 µM) or on receptors with a point mutation (F484A) in the GluN1 glycine-binding site that decreases glycine affinity (from EC_50_ = 0.52 µM to 3.3 mM)^56^. To ensure that contaminating glutamate was not a factor, we introduced a point mutation (H486A) in the GluN2B glutamate-binding site that decreases glutamate affinity (from EC_50_ = 1.6 µM to 0.52 mM)^71^. There was no significant difference between FRET histograms obtained in the presence and absence of added glutamate when the glycine-binding site was blocked by CGP or when glycine binding was prevented by the SNAP_GluN1-1a(F484A) mutation (Fig. 4b). Nor was there a difference in FRET histograms between wild-type SNAP_GluN1-1a/GluN2B in the absence of added glutamate and with glycine- binding site blocked by CGP corresponds and the combined GluN1 and GluN2 affinity site mutations SNAP_GluN1-1a(F484A)/GluN2B(H486A) (Fig. 4b). These observations show that we obtain the Apo state of the SNAP_GluN1-1a/GluN2B receptor in zero-added glutamate and 3 µM CGP.

### Confocal imaging

HEK293T cell imaging was performed on upright, scanning confocal microscope Zeiss LSM 780 (ZEN 2.3 acquisition software) with ×20 water immersion objective and 561-nm laser excitation.

### Statistical analysis

Unless otherwise mentioned, error bars represent SEM; statistical comparison of groups was performed using Student’s two-tailed *t* test (*P* < 0.05 was used to determine the significance) in SigmaPlot 10.

### Reporting summary

Further information on research design is available in the Nature Research Reporting Summary linked to this article.

## Supplementary information

Supplementary Information

Reporting Summary

## Data Availability

Data supporting the findings of this paper are available from the corresponding author upon reasonable request. A reporting summary for this article is available as a Supplementary Information file. Source data are provided with this paper.

## Source data

Source Data
