# Supplementary material for: Conformational rearrangement of the NMDA receptor amino-terminal domain during activation and allosteric modulation
Source: Nat Commun. 2021 May 11;12:2694. doi: 10.1038/s41467-021-23024-z (PMC8113580; doi:10.1038/s41467-021-23024-z)
Supplement: Supplementary file 1 — Supplementary Information [file 41467_2021_23024_MOESM1_ESM.pdf]

Supplementary information for:

Conformational rearrangement of the NMDA receptor amino-terminal domain during activation and allosteric modulation

Vojtech Vyklicky,[1,2] Cherise Stanley,[1] Chris Habrian,[3] and Ehud Y. Isacoff[1,3,4,5,*]

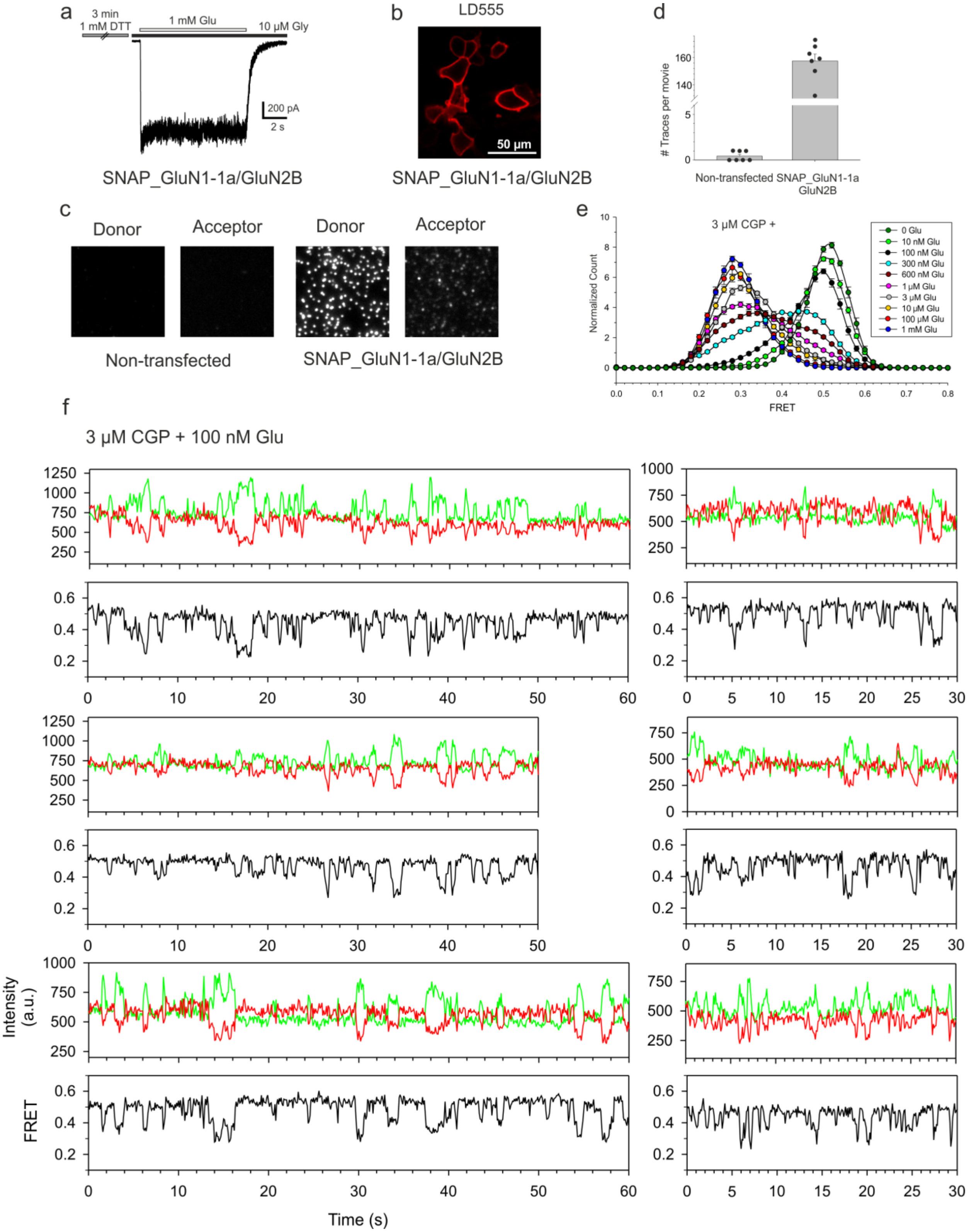
a
3 min
1 mM DTT
1 mM Glu
10 µM Gly
200 pA
2 s
SNAP_GluN1-1a/GluN2B
b
LD555
50 µm
SNAP_GluN1-1a/GluN2B
d
Traces per movie
Non-transfected
SNAP_GluN1-1a GluN2B
c
Donor
Acceptor
Donor
Acceptor
Non-transfected
SNAP_GluN1-1a/GluN2B
e
3 µM CGP +
Normalized Count
FRET
0 Glu
10 nM Glu
100 nM Glu
300 nM Glu
600 nM Glu
1 µM Glu
3 µM Glu
10 µM Glu
100 µM Glu
1 mM Glu
f
3 µM CGP + 100 nM Glu
Intensity (a.u.)
FRET
Time (s)

Supplementary Fig. 1: smFRET assay and glutamate-induced conformational change.

a, Representative current trace induced in fully functional SNAP_GluN1-1a/GluN2B receptor-expressing HEK293T cells by fast application of 1 mM glutamate (in the continuous presence of 10 μM glycine). Receptors were pretreated by 1 mM DTT in agonist free solution for 3 min immediately before agonist application. **b**, Confocal image of donor (LD555-BG) labeled HEK293T cells expressing SNAP_GluN1-1a/GluN2B receptors. This experiment was repeated more than 10 times with similar results. **c**, Antibody against the GluN1-1a C-terminal HA-tag and LD555-BG/LD655-BG SNAP-tag labeling approach leads to highly specific labeling and SimPull purification of NMDAR. **d**, Labeling and SimPull is highly specific. Quantification of FRET traces of non-NMDAR origin (n=7 movies, SEM error bars). **e**, FRET histograms recorded with increasing glutamate concentration in presence of 3 μM CGP (each concentration n=5 movies, SEM error bars). **f**, Representative donor (green) and acceptor (red) intensity traces and corresponding smFRET trace (black) in presence of 3 μM CGP and 100 nM glutamate.

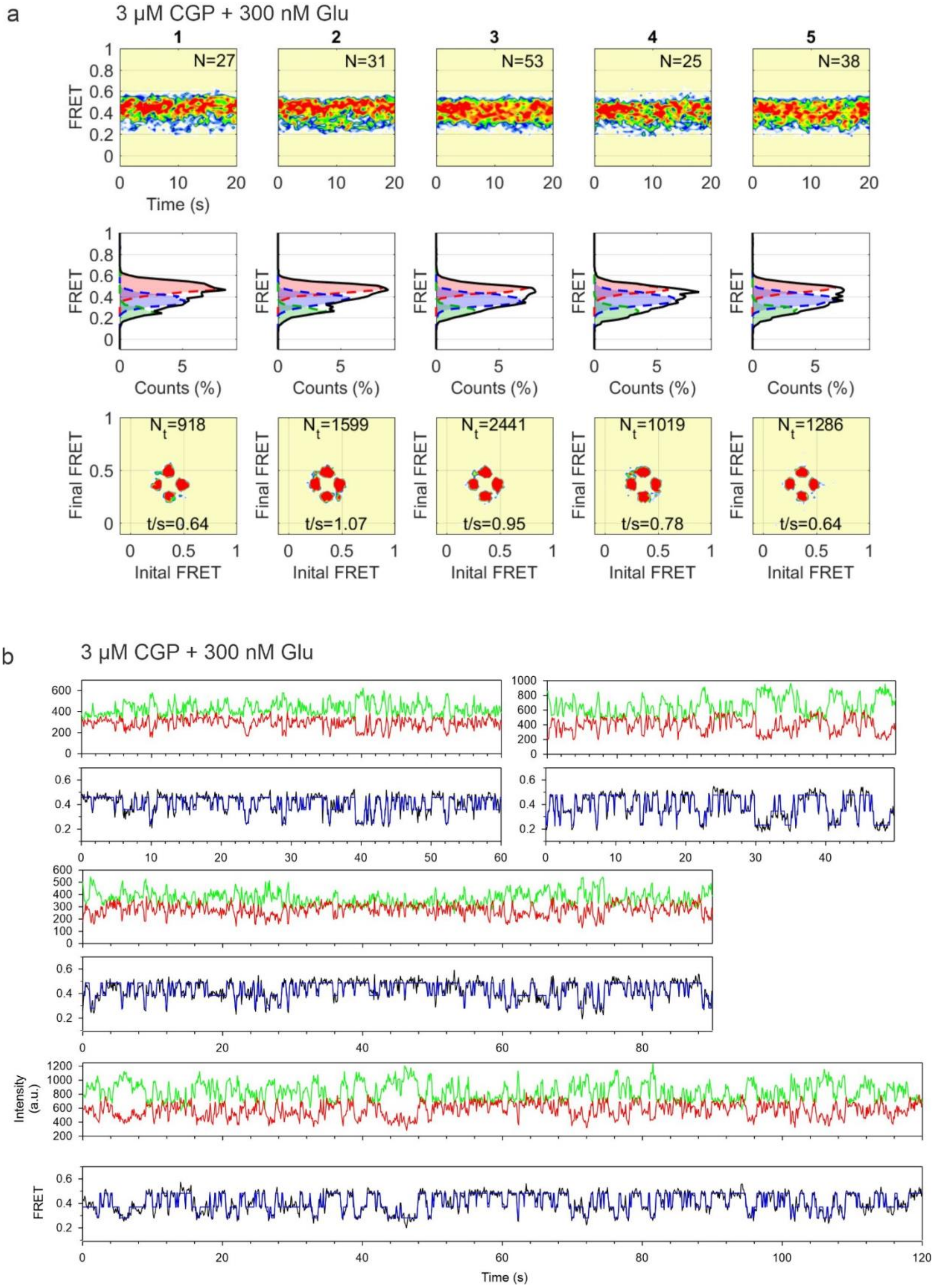
a
3 μM CGP + 300 nM Glu
1
2
3
4
5
N=27
N=31
N=53
N=25
N=38
FRET
Time (s)
Counts (%)
N_t=918
N_t=1599
N_t=2441
N_t=1019
N_t=1286
t/s=0.64
t/s=1.07
t/s=0.95
t/s=0.78
t/s=0.64
Final FRET
Inital FRET
b
3 μM CGP + 300 nM Glu
Intensity (a.u.)
FRET
Time (s)

Supplementary Fig. 2: Reproducibility across multiple movies.

a, Population FRET contour plots for receptors treated by 3 µM CGP and 300 nM glutamate (N number of molecules; top). Corresponding cumulative populations histograms of SKM identified states (middle). Transition density plots show FRET values before (initial) and after (final) each transition (t/s = averaged number of transitions per second; N_t = total transitions per movie; bottom). **b**, Representative donor (green) and acceptor (red) intensity traces and corresponding smFRET trace (black) with 3-state SKM idealization (blue) in 3 µM CGP and 300 nM glutamate. Each trace is from different movie.

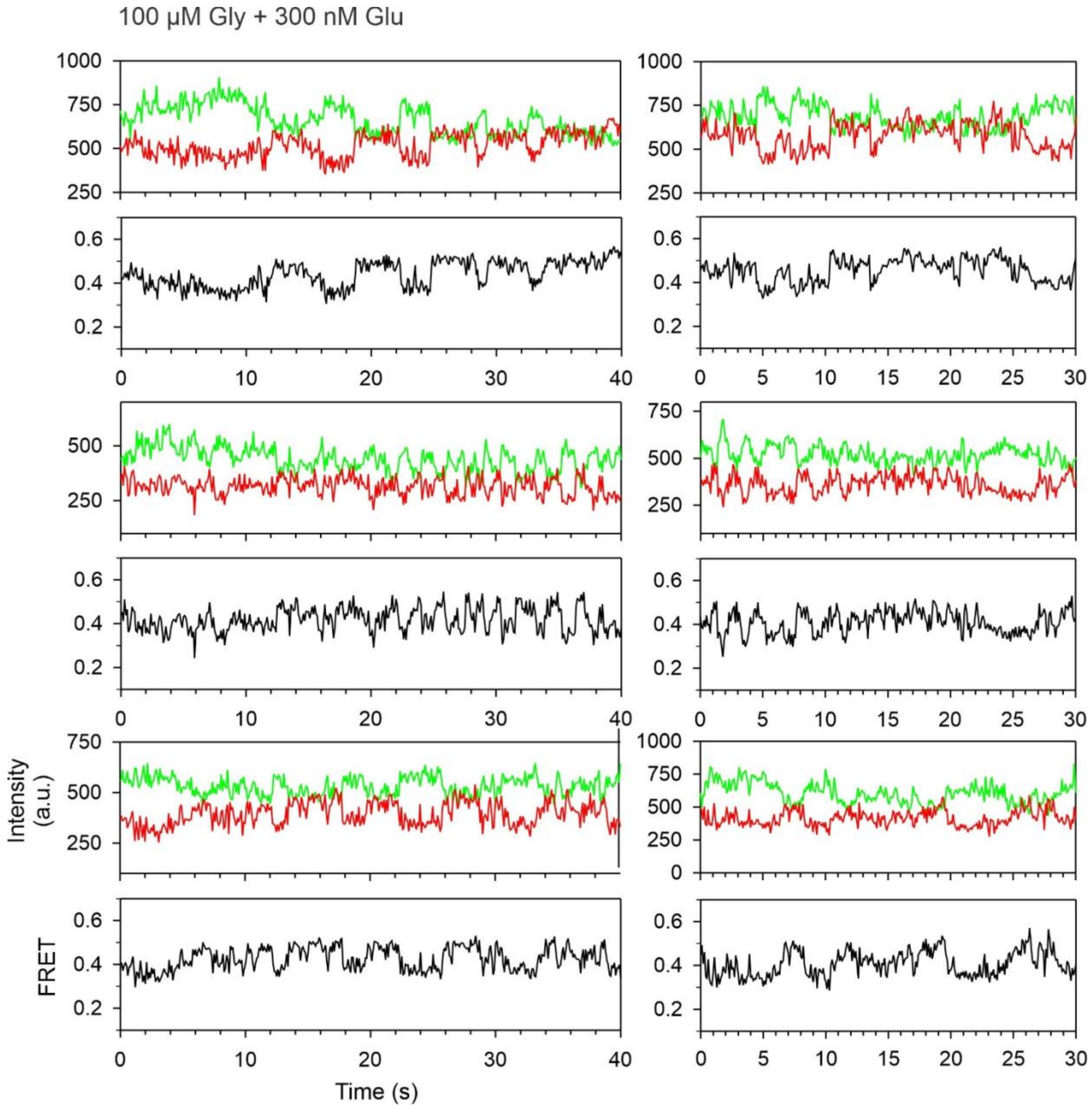

Supplementary Fig. 3: Representative donor (green) and acceptor (red) intensity traces and corresponding smFRET trace (black) in presence of 100 μM glycine and 300 nM glutamate.

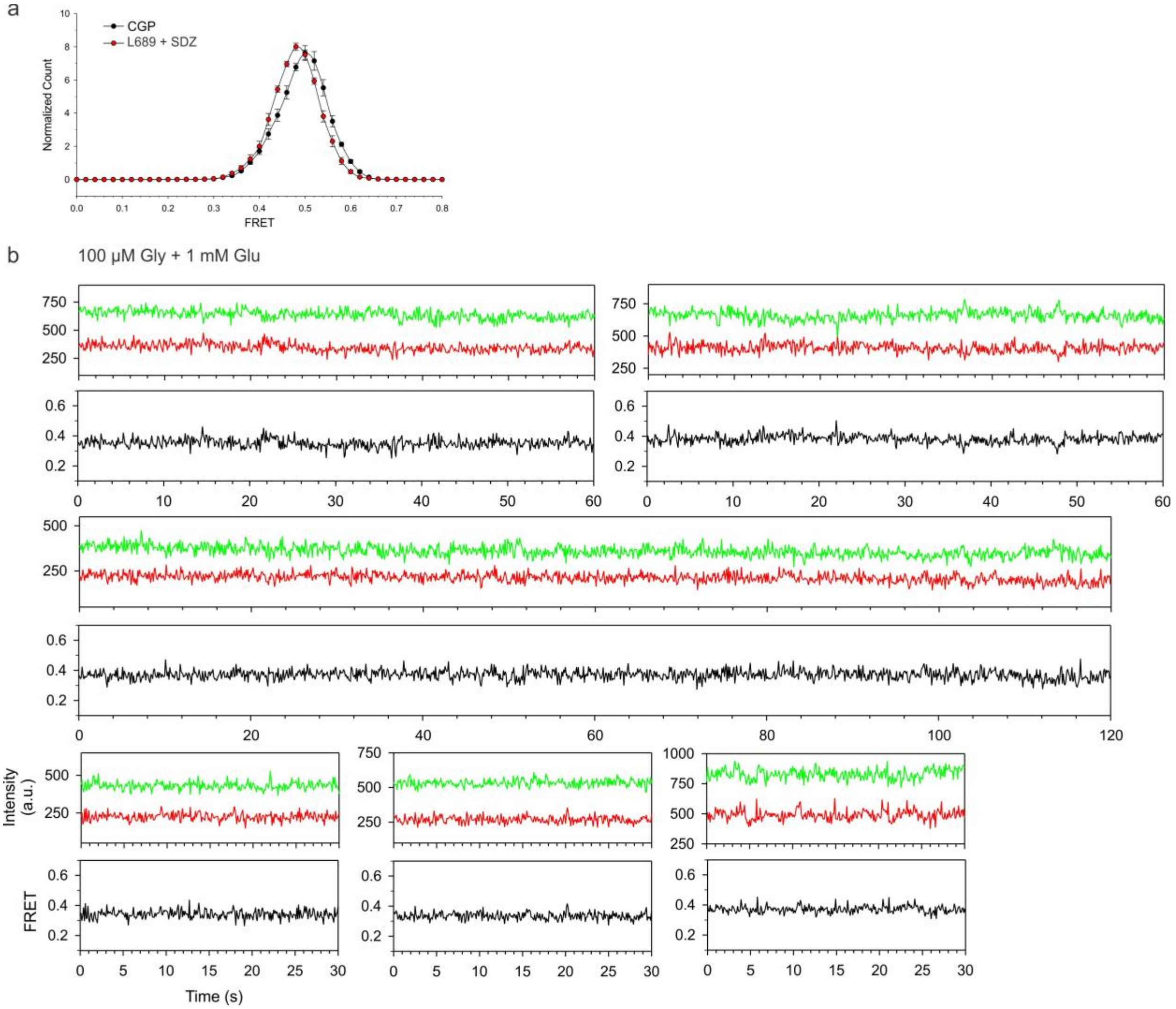

Supplementary Fig. 4:

a, Receptor conformation in presence of competitive antagonist 1 µM L689,560 (L689) and 50 µM SDZ-220-040 (SDZ) is significantly different from Apo state (3 µM CGP) at pH 8.5 (p=0.023, two-tailed t-test, n=5 movies, SEM error bars). **b**, Representative donor (green) and acceptor (red) intensity traces and corresponding smFRET trace (black) in presence of 100 µM glycine and saturating 1 mM glutamate.

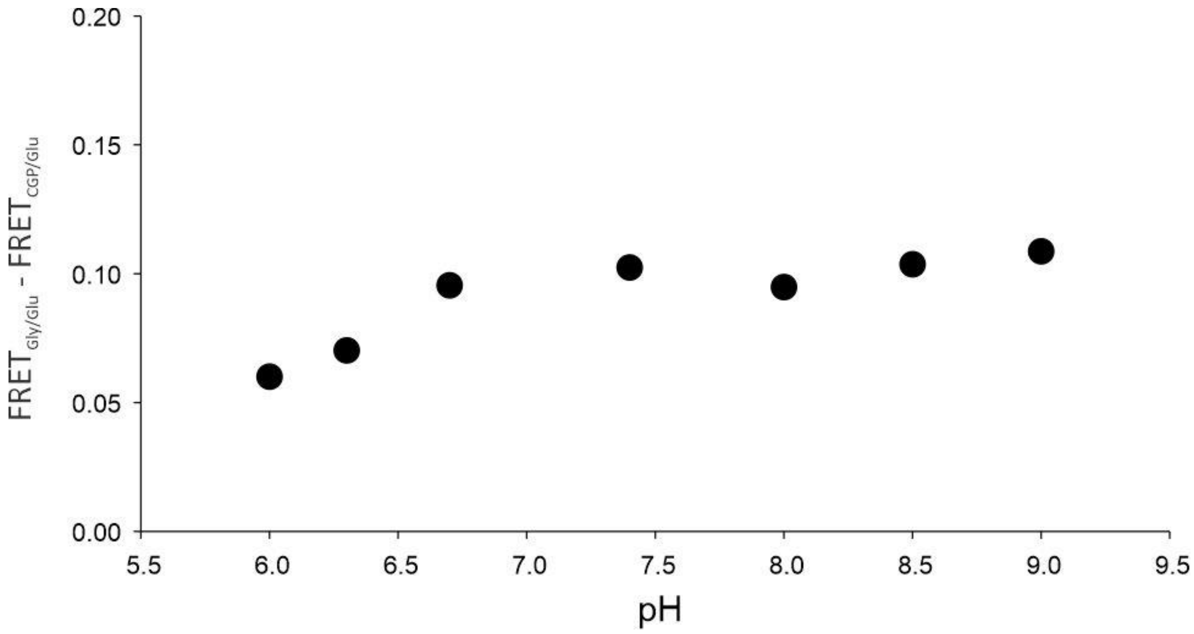

Supplementary Fig. 5: Shift in FRET due to addition of glycine is constant across a large pH range.
