# Supplementary material for: Conformational rearrangement of the NMDA receptor amino-terminal domain during activation and allosteric modulation
Source: Nat Commun. 2021 May 11;12:2694. doi: 10.1038/s41467-021-23024-z (PMC8113580; doi:10.1038/s41467-021-23024-z)
Supplement: Supplementary file 2 — Reporting Summary [file 41467_2021_23024_MOESM2_ESM.pdf]

nature research

Corresponding author(s): Ehud Y. Isacoff

Last updated by author(s): Mar 19, 2021

# Reporting Summary

Nature Research wishes to improve the reproducibility of the work that we publish. This form provides structure for consistency and transparency in reporting. For further information on Nature Research policies, see our Editorial Policies and the Editorial Policy Checklist.

## Statistics

For all statistical analyses, confirm that the following items are present in the figure legend, table legend, main text, or Methods section.

| n/a | Confirmed | |
|---|---|---|
| ☐ | ☒ | The exact sample size (*n*) for each experimental group/condition, given as a discrete number and unit of measurement |
| ☐ | ☒ | A statement on whether measurements were taken from distinct samples or whether the same sample was measured repeatedly |
| ☐ | ☒ | The statistical test(s) used AND whether they are one- or two-sided<br>*Only common tests should be described solely by name; describe more complex techniques in the Methods section.* |
| ☒ | ☐ | A description of all covariates tested |
| ☒ | ☐ | A description of any assumptions or corrections, such as tests of normality and adjustment for multiple comparisons |
| ☐ | ☒ | A full description of the statistical parameters including central tendency (e.g. means) or other basic estimates (e.g. regression coefficient) AND variation (e.g. standard deviation) or associated estimates of uncertainty (e.g. confidence intervals) |
| ☐ | ☒ | For null hypothesis testing, the test statistic (e.g. *F*, *t*, *r*) with confidence intervals, effect sizes, degrees of freedom and *P* value noted<br>*Give P values as exact values whenever suitable.* |
| ☒ | ☐ | For Bayesian analysis, information on the choice of priors and Markov chain Monte Carlo settings |
| ☒ | ☐ | For hierarchical and complex designs, identification of the appropriate level for tests and full reporting of outcomes |
| ☒ | ☐ | Estimates of effect sizes (e.g. Cohen's *d*, Pearson's *r*), indicating how they were calculated |

*Our web collection on statistics for biologists contains articles on many of the points above.*

## Software and code

Policy information about availability of computer code

| | |
|---|---|
| Data collection | MicroManager 2.0 gamma (smFRET), pClamp 10 (patch-clamp), ZEN 2.3 (live cell confocal imaging) |
| Data analysis | SPARTAN 3.7.0. (smFRET), QuB 1.4 (smFRET), Fityk 1.3.1. (smFRET), pClamp 10 (patch-clamp), SigmaPlot 10 (statistics) |

For manuscripts utilizing custom algorithms or software that are central to the research but not yet described in published literature, software must be made available to editors and reviewers. We strongly encourage code deposition in a community repository (e.g. GitHub). See the Nature Research guidelines for submitting code & software for further information.

## Data

Policy information about availability of data

All manuscripts must include a data availability statement. This statement should provide the following information, where applicable:
- Accession codes, unique identifiers, or web links for publicly available datasets
- A list of figures that have associated raw data
- A description of any restrictions on data availability

Data supporting the findings of this manuscript are available from the corresponding author upon reasonable request and as a Supplementary Source Data file.

# Field-specific reporting

# Life sciences study design

All studies must disclose on these points even when the disclosure is negative.

| | |
|---|---|
| Sample size | smFRET histogram sample size ranged from 46 to 446 individual molecules (all single molecule traces were at least 20 s long = 200 data points) from at least 4 movies. We have estimated that minimal sufficient sample size for smFRET histogram is ~8000 data points. Additional increase in the number of data points did not have significant effect on final histogram parameters. |
| Data exclusions | No data was excluded. |
| Replication | All experiments were repeated at least three times on multiple days with similar results. |
| Randomization | The individual NMDAR receptor ligands were applied and recorded in random order. |
| Blinding | smFRET experiments are not subjected to experimenter bias. All smFRET imaging data recorded in presence of different ligands were automatically analyzed by software using identical settings. |

# Reporting for specific materials, systems and methods

We require information from authors about some types of materials, experimental systems and methods used in many studies. Here, indicate whether each material, system or method listed is relevant to your study. If you are not sure if a list item applies to your research, read the appropriate section before selecting a response.

## Materials & experimental systems

| n/a | Involved in the study | |
|---|---|---|
| ☐ | ☒ | Antibodies |
| ☐ | ☒ | Eukaryotic cell lines |
| ☒ | ☐ | Palaeontology and archaeology |
| ☒ | ☐ | Animals and other organisms |
| ☒ | ☐ | Human research participants |
| ☒ | ☐ | Clinical data |
| ☒ | ☐ | Dual use research of concern |

## Methods

| n/a | Involved in the study | |
|---|---|---|
| ☒ | ☐ | ChIP-seq |
| ☒ | ☐ | Flow cytometry |
| ☒ | ☐ | MRI-based neuroimaging |

## Antibodies

| | |
|---|---|
| Antibodies used | Anti-HA tag antibody (Biotin) (ab26228) |
| Validation | Antibody specificity was validated on non-transfected HEK293T cells by SimPull method. |

## Eukaryotic cell lines

Policy information about cell lines

| | |
|---|---|
| Cell line source(s) | HEK293T, ATCC |
| Authentication | No |
| Mycoplasma contamination | Cell cultures not tested for mycoplasma contamination. |
| Commonly misidentified lines (See ICLAC register) | No commonly misidentified cell line was used in the study. |
